# N-Acetyl L-Cysteine does not protect mouse ears from the effects of noise*

**DOI:** 10.1186/1745-6673-5-11

**Published:** 2010-04-28

**Authors:** Rickie R Davis, David A Custer, Edward Krieg, Kumar Alagramam

**Affiliations:** 1 Hearing Loss Prevention Team, Engineering and Physical Hazards Branch, Division of Applied Research and Technology, National Institute for Occupational Safety and Health, C-27, 4676 Columbia Parkway, Cincinnati, OH 45226, USA; 2 Department of Biological Sciences, University of Cincinnati, Cincinnati, OH, USA; 3 Statistics Team, Division of Applied Research and Technology, National Institute for Occupational Safety and Health, C-27, 4676 Columbia Parkway, Cincinnati, OH 45226, USA; 4 Department of Otolaryngology-HNS, University Hospitals Case Medical Center, Case Western Reserve University, Cleveland, OH, 44106 USA

## Abstract

**Background:**

Noise-induced hearing loss (NIHL) is one of the most common occupational injuries in the United States. It would be extremely valuable if a safe, inexpensive compound could be identified which protects worker hearing from noise. In a series of experiments, Kopke has shown that the compound N-acetyl-L-cysteine (L-NAC) can protect the hearing of chinchillas from the effects of a single exposure to noise. L-NAC is used in clinical medicine and is very safe. Although L-NAC was reported to be promising, it has not been successful in other studies (Kramer et al., 2006; Hamernik et al., 2008). The present study was undertaken to determine if L-NAC could protect C57BL/6J (B6) mice from the permanent effects of noise.

**Method:**

Two groups of five B6 mice were injected with either 300 or 600 mg/kg L-NAC approximately 1 hr prior to a 104 dB broadband noise exposure and again immediately after the exposure. A control group (N = 7) was exposed to the same noise level but injected with vehicle (sterile saline). Auditory brainstem response measurements were made at 4, 8, 16 and 32 kHz one week prior to and 12 days after exposure.

**Conclusions:**

There were no statistically significant differences in ABR threshold shifts between the mice receiving L-NAC and the control mice. This indicates that L-NAC was not effective in preventing permanent threshold shift in this mouse model of NIHL.

## Background

The inbred mouse strain C57BL/6J (B6) has been shown to be more susceptible to noise-induced hearing loss (NIHL) than the CBA/CaJ strain [1]. This phenotype has been traced to a mutation of the gene coding for cadherin 23, C*dh23 *[2].

Staecker et al. [3] demonstrated that the antioxidant systems of B6 mice have a number of differences when compared with normal-hearing strains (i.e. CBA/CaJ and a congenic B6 strain (B6.CAST+ahl mouse) with the *Ahl *allele replaced with the wild-type *Castaneous *strain allele). Using immunohistochemical techniques they showed that qualitative levels of super-oxide dismutase, glutamyl transferase and 4-hydroxynonenal increase between 3 month old and 9 month old B6 mice; and differ from the levels detected in age-matched, normal hearing CBA/CaJ mice. Using semi-quanitative PCR analyses, levels of messenger RNA for copper/zinc and magnesium super-oxide dismutase and catalase B6 mice were statistically greater than levels expressed in 3 month old CBA/CaJ mice. On the other hand, the level of glutathione peroxidase did not differ statistically in the two strains. Based on Staecker's results one could argue that the oxidative stress system of a B6 mouse ear is not impaired by the *Cdh23 *mutation and in some cases may be enhanced. This contradicts evidence that B6 ears are more sensitive to noise-induced hearing loss [4].

One possible prophylactic agent against noise could be N-acetyl L-cysteine (L-NAC). L-NAC is extremely safe and has been used for many years to protect the liver from the toxic effects of acetaminophen overdose. L-NAC interacts directly with free radicals to prevent liver damage.

L-NAC (at 325 mg/kg) has been shown in chinchillas to protect the cochlea from the damaging effects of noise when combined with salicylate (at 50 mg/kg) and injected prior to noise exposure [5]. In chinchillas Bielefeld et al. [6] demonstrated protection by L-NAC to high kurtosis stimuli (at 325 mg/kg i.p.); protection at doses as low as 50 mg/kg (i.p.); and protection when given by oral gavage (325 mg/kg.) In contrast, a more recent study using L-NAC alone (325 mg/kg) did not detect functional or anatomical protection of the chinchilla inner ear to high kurtosis stimuli [7]. While it is possible that this outcome was related to the impulse noise insult selected for that study, Duan et al. [8] found dose-dependant protection of the inner ear in rats exposed to impulse noise. The best protection was obtained using a three times per day dose (350 mg/kg/injection). Animals that received only 1 injection per day, or 5 injections per day, received less benefit.

Kopke and collegues' double-blind, placebo controlled study of protective effects of oral L-NAC in U.S. Marines prior to firearms training is of great interest [9]. They report "a favorable biological response" on hearing in marines treated with L-NAC prior to small arms fire.

There have been three pathways proposed for the action for L-NAC. First, L-NAC is a precursor for glutathione, the body's natural reactive oxygen species (ROS) scavenger. Presumably, infusion of L-NAC increases the cochlea's store of glutathione. It has been shown in brain that glutathione is actively transported across the blood brain barrier by a saturable system while L-NAC is transported by a more general amino acid system. Presumably the same transporter systems are present in the cochlea [10]. Second, L-NAC has been shown to have basic protective properties independent of glutathione: both L- and D-isomers of NAC were able to protect cells *in vitro *from ROS. Since only the L isomer of NAC is enzymatically converted to glutathione, this strongly suggests that the protective effects of NAC can be independent of glutathione, probably through cell cycle regulation [11]. Third, in cell culture, L-NAC has been shown to block apoptosis probably through inducing specific gene expression [12].

Our hypothesis is that the administration of L-NAC should provide protection against noise sensitivity in B6 mice by boosting the free-radical scavenging mechanisms of the cochlea. Two dosages were chosen: one which has been shown to be protective in chinchillas and a second dose double the first. A protective effect should be evident.

## Materials and methods

All animal procedures were approved by the University of Cincinnati Institute Animal Care and Use Committee. Eighteen, four-week old female mice of the C57BL/6J strain were purchased from The Jackson Lab (TJL), Bar Harbor, ME. The mice were divided into three groups. The low dose group received an i.p. injection of 300 mg/kg of L-NAC (Sigma-Aldrich, Inc. #A7250, CAS 616-91-1) dissolved in sterile saline one hour before and immediately after noise exposure (N = 6). The high dose group received an i.p. injection of 600 mg/kg of L-NAC in sterile saline, adjusted to pH 7.0 by addition of sodium hydroxide, one hour before and immediately after noise exposure (N = 5). The pH was adjusted after high mortality was noted in an earlier high dose group. The control group received an equal volume of sterile saline one hour before and immediately after noise exposure (N = 7).

Auditory Brainstem Response. Mice were allowed to accommodate to the facility for one week. All mice were tested for the ability to generate the auditory brainstem response (ABR) in week two. Mice were anesthetized with an i.p. injection of Avertin (tribromoethanol, 0.4 mg/g). Twelve days after exposure mice were ABR tested for a second time.

Auditory brainstem responses (ABR) were generated to 4, 8, 16 and 32 kHz tone pips (tested in ascending order). Tone pips consisted of a three millisecond envelope: 1 ms ramp onset, 1 ms plateau and 1 ms decay. Tone pips were generated by Tucker-Davis Technologies System 2 hardware (Alachua, FL, USA) running BioSig^® ^Software on a Pentium class computer. The tone burst was presented binaurally through specula attached to supertweeters. The ABR was recorded through Grass^® ^stainless steel needle electrodes placed subcutaneously at the vertex (active), right cheek (inverting) and left cheek (common). The resulting signal was band-pass filtered (100-3000 Hz), amplified (10,000×) and digitized by a TDT Bioamp. Responses were collected and averaged at 30 presentations per second for up to 512 times. The stimulus was presented at 100 dB SPL and progressed downward in 5 dB steps until no response was identifiable. Presentations were halted early if the characteristic ABR was noted. A second trace was collected and compared with the first if there was some question if a response was recorded.

Tone bursts were calibrated by extending the tone burst plateau to one minute and measuring the output of the speakers via an 1/8" Brüel & Kjær (B&K) microphone and a Brüel & Kjær 2608 Measuring Amplifier. A short piece of polyethylene tubing was connected between the speculum of the supertweeter and the 1/8" microphone, similar to the technique described by Pearce et al. [13]. The microphone was calibrated by a B&K microphone calibrator.

Noise exposure. Mice were placed in a multi-compartment mesh cage for simultaneous exposure. Mice were exposed in the third week for one hour to a 104 dB SPL broadband noise (spectrum was published in Erway et al, 1996)[4]. This level was chosen to produce a measurable threshold shift in B6 mice but not a total hearing loss [1]. The exposure conditions were continuously monitored by a 1/4" microphone attached to a Brüel & Kjær 2608 Measuring Amplifier.

Statistical Methods. Analysis of variance (ANOVA) was used to test for differences in ABR thresholds between the three groups pre-exposure and post-exposure as well as threshold shift due to exposure. A separate model was used for each frequency. Pairwise contrasts were done if an effect in an exposure group was statistically significant (p < 0.05). All calculations were done with SAS (Version 9.2, SAS Institute, Cary, NC).

## Results

**Figure 1:**
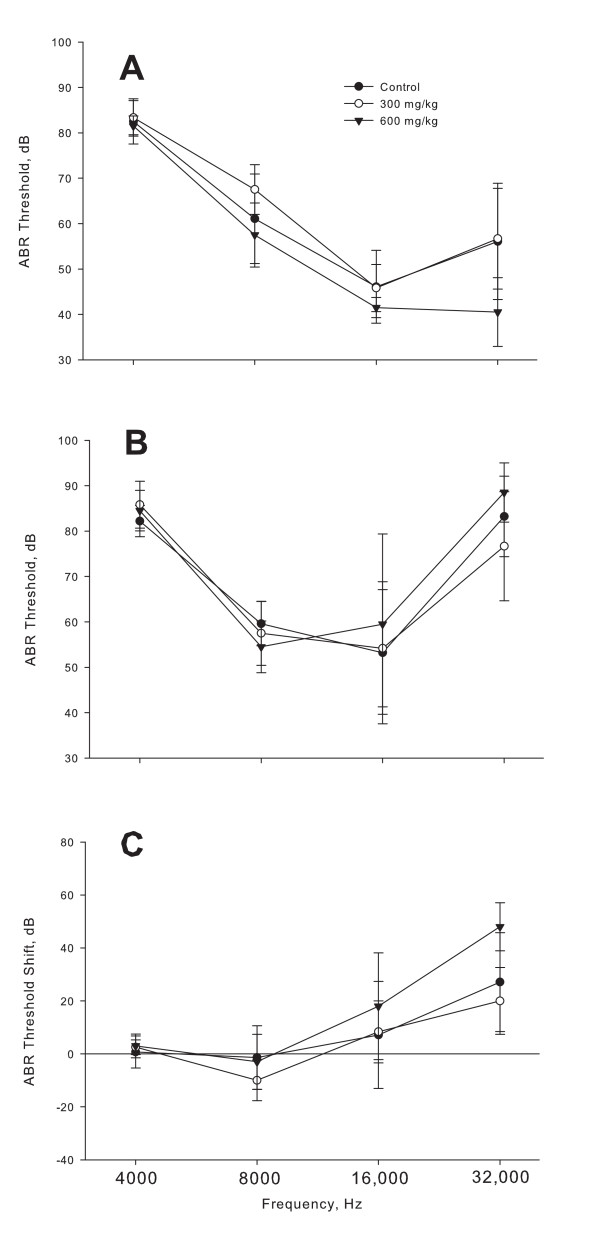
**Mean Auditory Brainstem Response (ABR) thresholds in decibels (dB) for 4, 8, 16 and 32 kHz for C57BL/6J mice**. (A) Pre-exposure ABR thresholds. (B) Twelve day post-exposure thresholds. (C) Threshold shifts, post-threshold minus pre-threshold. Error bars indicate ± 1 standard deviation.

The analysis of variance found no statistically significant protective effect for L-NAC. A protective effect would be demonstrated by a decrease in threshold shift with increasing L-NAC dose (0, 300 mg/kg, 600 mg/kg). This was not seen (Figure 1c). Post-exposure ABR thresholds did not differ in the three groups (Figure 1b; 4 kHz, F = 0.58, p = 0.57; 8 kHz, F = 1.11, p = 0.36; 16 kHz, F = 0.24, p = 0.79; 32 kHz, F = 2.13, p = 0.15).

At 32 kHz, prior to noise exposure the 600 mg/kg group mean was statistically significantly better than the two other group means. We determined that this was probably due to two mice who had stellar ABR thresholds at 32 kHz--5 to 10 dB better than their peers. (The pre-exposure mean thresholds were: control group 56.1 dB, the low dose group was 56.7 dB and the high dose group was 40.5 dB). The high dose group mean ABR thresholds after exposure were about the same as the rest of the groups (88.5 dB vs 83.2 dB for the control and 76.6 dB for the low dose group) but it also affected threshold shift for that frequency. The mean threshold shift at 32 kHz for the high dose group (48 dB) was greater than for the control (27.1 dB) or low dose group (20 dB).

## Discussion

The present data demonstrate that L-NAC does not protect B6 mouse hearing from moderate noise exposure. Our prediction that an ROS scavenger, L-NAC, would protect the hearing of the mice was not upheld.

We believe that the failure to protect the C57BL/6J mice may be related to one of the following: Dose level, a non-ROS mechanism of B6 noise-induced hearing loss, or a species specific lack of effect by L-NAC. These hypotheses provide directions for further research.

First, the dosage levels of 300 mg/kg and 600 mg/kg were chosen based on previous studies in chinchillas showing a dose of 325 mg/kg as protective against NIHL. We chose dosages that should be within the protective range. Generally, dosages must be adjusted up in smaller animals to obtain equivalent effect. It is possible that in the mouse an even higher dose of L-NAC might be necessary for otoprotection but we saw no evidence of *any *protection in the present study. Bliefield et al. [6] demonstrated some protection in chinchillas even at 50 mg/kg. Even if the mouse required a dose a magnitude larger than the chinchilla, a 600 mg/kg dose would meet that criterion.

Second, it is possible that ROS damage is secondary to the stereocillia defect in affecting noise-induced hearing loss in these mice. More likely, however, is that B6 mice have weakened hair cells due to the abnormal *Cdh23 *defect. Cadherin 23 is believed to make up part of the stereocillia tip-links. The dysfunctional hair cells in this strain make them particularly vulnerable to environmental insults or age-related hearing loss. We argue that B6 are an excellent model for NIHL, especially modeling of susceptible individuals.

Moreover, Staecker et al. [3] have shown production of endogenous antioxidants in the B6 inner ear. They compared age-matched B6 with normal hearing CBA/CaJ mice and noted increased labeling for super-oxide dismutase, glutamyl transferase and 4-hydroxynonenal in the lateral wall and spiral ganglia of B6 mice, which varied with age between 3 and 9 months of age. Expression levels of antioxidant enzyme mRNA were also elevated, with peak expression varying with age. Levels of messenger RNA for copper/zinc and magnesium super-oxide dismutase increased gradually up to 9 months of age, and were greater than levels expressed in 3 or 9 month old CBA/CaJ mice. Catalase expression peaked at 6 months of age in the B6 mice, but was much higher at 9 months of age in the CBA/CaJ group. On the other hand, the level of glutathione peroxidase did not differ statistically from the CBA/CaJ baseline. The time course of these changes parallels the hearing threshold shifts and cochlear degeneration which begin at 3-6 months of age. Although cochlear degeneration and hearing loss in B6 mice was originally attributed to the *Ahl *locus harboring the cadherin 23 mutation, the picture is now more complicated.

Finally, it is possible that mice may not be protected by L-NAC. First, L-NAC showed no protection again age-related hearing loss in mice [14]. Blakley et al. [15] reviewed differences between species highlighting the differences in ototoxic dose for gentamicin and cisplatin between mice and guinea pigs. Le Prell et al. [16] demonstrated that a vitamin and mineral antioxidant regime protected CBA/J mice from NIHL. Pharmacokinetic measurements of L-NAC and/or glutathione in mouse cochlear perilymph and tissue would be very useful but technically challenging to accomplish.

Two recent studies have shown no otoprotective effect for L-NAC. Kramer et al. [17] showed that L-NAC did not protect young adults from temporary threshold shift while attending a disco venue. Hamernik et al. [7] demonstrated that L-NAC did not protect the hearing of chinchillas when used in conjunction with high kurtosis impulse noise.

A safe, inexpensive, oral compound which displays prophylactic protection against noise in humans would be welcomed. Current efforts are underway to study otoprotectant antioxidants in human cohorts. Le Prell et al.[18] have identified a mix of vitamins and minerals effective at preventing NIHL in guinea pigs. Their mixture is currently undergoing human clinical trials to determine effectiveness. Campbell et al. [19] have investigated the use of D-methionine as an otoprotectant against both noise and ototoxic drugs. They, too, are clinically testing their compound on human populations. Ebselin has been identified as an otoprotectant in animal models [20] and is undergoing human trials. A quick search of the literature databases identify a number of compounds which have been implicated for otoprotection but have not been further developed.

A compound which protects hearing against both aging and noise damage would be doubly welcome. Although research is active, presently there are no compounds identified which meet these needs. Currently, the effective use of hearing protection devices appears to be the best defense against noise-induced hearing loss.

## Conclusions

Our data demonstrate that L-NAC does not protect B6 mouse hearing from moderate noise exposure. Our prediction that an ROS scavenger, L-NAC, would protect the hearing of the mice was not upheld. We conclude that it is premature to recommend that ROS scavengers be substituted for noise reduction or hearing protection for protecting worker's hearing.

## Abbreviations

ABR: auditory brainstem response; ANOVA: analysis of variance; B6: the C57BL/6J strain of mouse; *Cdh23*: gene coding for cadherin 23 protein, equivalent to *Ahl*; dB SPL: decibels referenced to 20 μN per meter^2^; i.p.: intraperitoneal; L-NAC: N-acetyl-L-cysteine; NIHL: noise induced hearing loss; PCR: polymerase chain reaction; ROS: reactive oxygen species.

## Competing interests

The authors declare that they have no competing interests.

## Authors' contributions

RRD and KA conceived and designed the study. RRD and DC conducted the study. EK conducted statistical analysis of the collected data. All authors contributed to the writing of the manuscript. All authors have read and approved the final manuscript.
